# Playing a musical instrument increases blood flow in the middle cerebral artery

**DOI:** 10.1371/journal.pone.0269679

**Published:** 2022-06-08

**Authors:** Ai Kawasaki, Naoyuki Hayashi

**Affiliations:** 1 Department of Social and Human Sciences, Tokyo Institute of Technology, Tokyo, Japan; 2 Faculty of Sport Sciences, Waseda University, Saitama, Japan; University of Caytania, ITALY

## Abstract

**Purpose:**

Studies using functional magnetic resonance imaging and positron-emission tomography suggest that many regions of the brain are activated by such complex muscle activity. Although these studies demonstrated relative increases in blood flow in some brain regions with increased neural activity, whether or not the absolute value of cerebral blood flow increases has yet to be elucidated. It also remains unknown whether playing musical instruments affects cerebral blood flow. The aim of this study was to determine the impact of playing a musical instrument on blood flow velocity in the middle cerebral artery (MCAv) by using Doppler ultrasound to measure absolute values of arterial flow velocity.

**Methods:**

Thirteen musicians performed three pieces of music with different levels of difficulty: play for the first time (FS), music in practice (PR) and already mastered (MS) on either piano or violin. MCAv was recorded continuously from 10 min before until 10 min after playing. Associations between the cerebral blood flow response and blood pressure and gas-exchange variables were examined.

**Results:**

PR and MS significantly increased the MCAv. The blood pressure increased significantly in performances of all difficulty levels except for MS. There were no significant changes in exhaled gas variables during the performance.

**Conclusion:**

These findings suggest that playing a musical instrument increases MCAv, and that this change is influenced by the difficulty of the performance.

## Introduction

Many regions of the brain are activated when listening to music or playing a musical instrument, as demonstrated by studies using functional magnetic resonance imaging (fMRI) and positron-emission topography (PET) [1–4]. This activation is identified by increases in blood volume relative to other brain regions. However, neither fMRI nor PET can provide information regarding absolute changes in blood volume or blood flow. Global cerebral blood volume and cerebral blood flow (CBF) can be stable if increases in blood volume and flow in activated regions of the brain are offset by slightly decreased in blood volume and flow in other regions that are not activated. The effect of playing a musical instrument on absolute changes in global CBF has yet to be determined.

Playing a musical instrument can increase global CBF. Middle cerebral artery (MCA) blood velocity (MCAv), which reflects a wide range of CBF, increases by up to 20% with physical activities such as cycling exercise by [5]. Playing a musical instrument has a much lower metabolic burden [6, 7] but is associated with more complex muscle activity than such physical activity. Complex movements require an interaction between local activation and brain interregional sensorimotor communication. Moreover, complex motor tasks involve larger regions of the brain with increased regional CBF than do simple motor tasks [8]. Thus, playing a musical instrument can increase CBF as much as physical activity.

Transcranial Doppler (TCD) flowmetry is a non-invasive method of assessing absolute changes in blood flow velocity in large arteries, such as the MCA in humans, in various situations and at a high sampling rate [9, 10]. TCD is inexpensive, safe, and useful for intracranial pressure monitoring [11]. It is generally accepted that the CBF measured by TCD reflects the change in global CBF. It has been demonstrated that during exercise, the change in MCAv measured by TCD is comparable to the change in mean blood flow in its upstream artery, the internal carotid artery [5]. TCD can record cerebral blood flow while musicians play music in normal circumstances. Also, PET and fMRI produce noise, whereas TCD generates no sound during the examination. Subjects can hear normal performance feedback of the sound they make.

The present study used TCD flowmetry to determine the absolute change in MCAv in participants while they were playing a musical instrument. The contributions of candidate factors underlying changes in CBF were explored, including blood pressure (BP) and arterial carbon dioxide partial pressure (PaCO_2_), which are known to alter CBF [12]. We hypothesized that CBF increases when playing a musical instrument, as it does during exercise.

## Methods

### Subjects

A cohort of 13 musicians who had been playing musical instruments for more than 6 years was recruited. The subjects had no history of autonomic nervous system disease or heart disease, and had not smoked or taken medication. There was no history of cognitive impairment or depression in the participants. They provided written consent to participate in this study after receiving an explanation regarding its purpose and what their involvement would be. This study was approved by the Human Subjects Research Ethics Review Committee of Tokyo Institute of Technology (No. 2017098). This study was conducted according to the guidelines laid down in the Declaration of Helsinki

### Experimental protocol

On the day of the experiment, subjects were asked to refrain from taking caffeine and strenuous exercise for 6 hours before the experiment, and from eating for 2 hours before the experiment. The subjects were asked to play three music scores with three different levels of difficulty for 10 min in a randomized order: music for seen for the first time (FS), music in practice (PR) and music already mastered (MS). The subjects were asked to provide in advance the FS, PR and MS scores. The music score for the FS was confirmed before the experiment that the subject was playing it for the first time. A rest period of at least 10 min was provided between performances.

MCAv was measured continuously from 10 min before to 10 min after the performances. BP was measured immediately before the performance and when it had ended. At minute 9 of the 10-min performance, a cuff for BP measurement was attached to the subject’s right arm. Respiration data were measured for 1 min in six subjects simultaneously with the BP measurement.

### Measurements

MCAv was measured using a TCD flow meter (WAKI, Atys Medical, St-Genis-Leval, France) via a probe applied to the left temporal region with a headband. BP was measured by a upper arm sphygmomanometer (UA-704, A&D, Tokyo Japan). Tidal volume and end-tidal partial pressure of carbon dioxide (P_ET_CO_2_) were measured using a gas analyser (AE-310S, Minato Medical Science, Osaka Japan) on a breath-by-breath basis via a mouthpiece and nose clip.

### Data analysis

Data are expressed as mean± SD values. MCAv and P_ET_CO_2_ for the last minute of each rest, playing and recovery phase of every performance were averaged and used for analysis. The cerebrovascular conductance index (Cl) was calculated by dividing the mean value of MCAv by the mean BP (MBP). CI is the ease with which blood flows when circulating at a given pressure differential, and conductance (blood flow/pressure) is the reciprocal of resistance (pressure/blood flow) [13]. The effect of time on MCAv and BP was examined by one-way repeated analysis of variance. When a significant *F* value was obtained, Dunnett’s *post hoc* test was used to compare rest, playing and recovery values. The level of statistical significance was set at *P*≤0.05. A standard statistical package (IBM SPSS Statistics 21.0 for Windows, IBM, Tokyo, Japan) was used for all statistical analyses.

## Results

The 13 musicians who participated in this study were 3 males and 10 females aged 30±11 years, among whom 3 were violinists and 10 were pianists.

**Fig 1:**
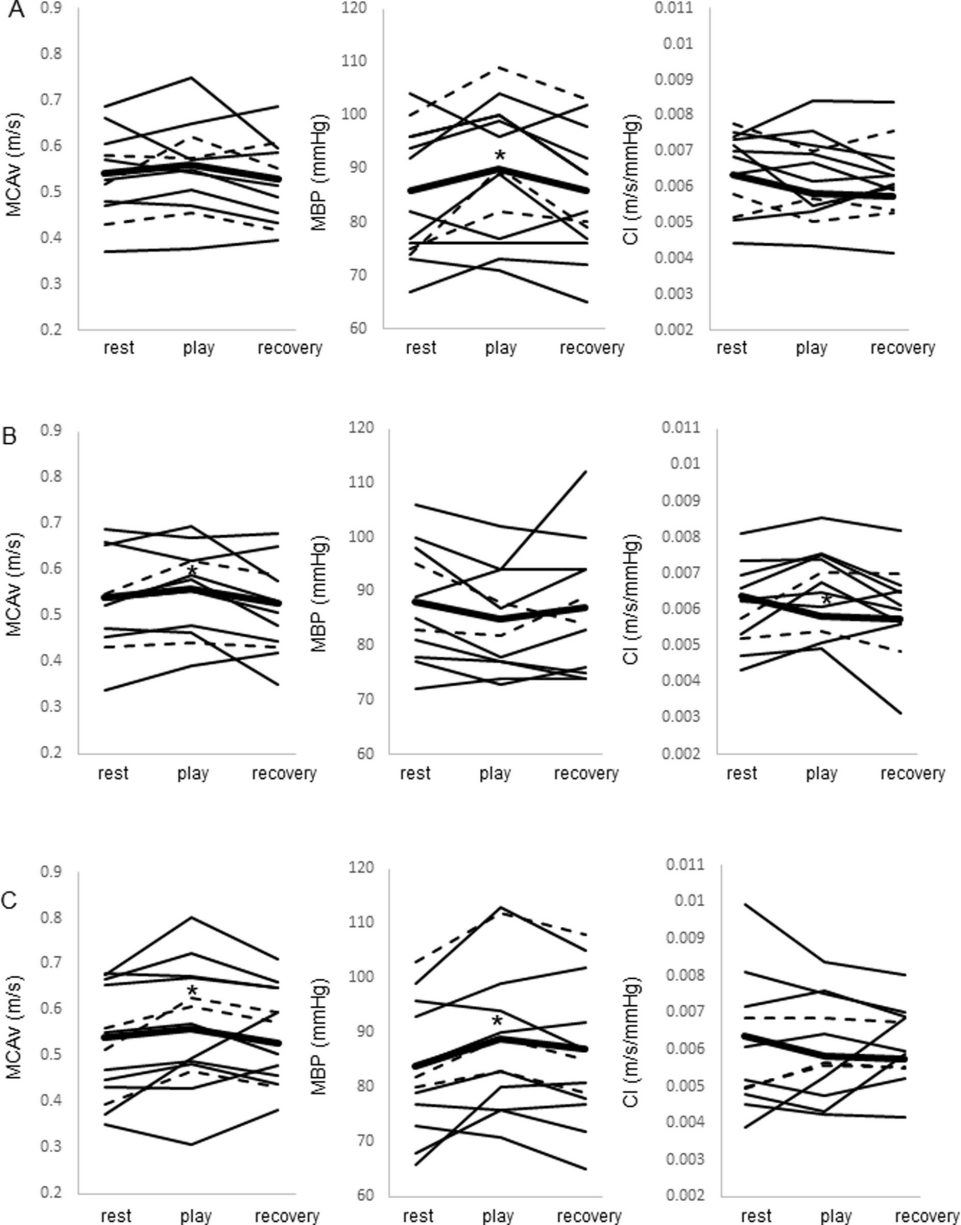
Mean values for all subjects, and individual values for piano players and violin players. Middle cerebral artery blood flow velocity (MCAv), mean blood pressure (MBP) and conductance of MCAv (CI) before, during and after playing a musical instrument in (A) the first time look (FS) trial, (B) music already mastered (MS) trial and (C) music in practice (PR) trial. Shown are mean values for all subjects (thick black lines), and individual values for piano players (thin black lines) and violin players (dotted lines). *P≤0.05 vs. resting.

Resting MBP values were very similar when playing, irrespective of the difficulty of the score that was about to be played, being 86±12, 88±10 and 84±11 mmHg for the FS, MS and PR trials, respectively (Fig 1). Significant increases in MBP were observed in the FS (90±13 mmHg) and PR (89±13 mmHg) trials, but not in the MS trial.

There was also no significant difference in resting MCAv in subjects before playing the FS, MS and PR scores (0.54±0.09, 0.53±0.1 and 0.52±0.1 m/s, respectively; Fig 1). MCAv significantly increased when playing the MS (0.55±0.09 m/s, 8.6%) and PR (0.56±0.14 m/s, 5.2%) scores, but not when playing the FS score.

The only significant change in CI was observed when playing the MS score, when there was a significant decrease. P_ET_CO_2_ and oxygen consumption (*V*O_2_) showed no significant changes with the score difficulty.

**Fig 2:**
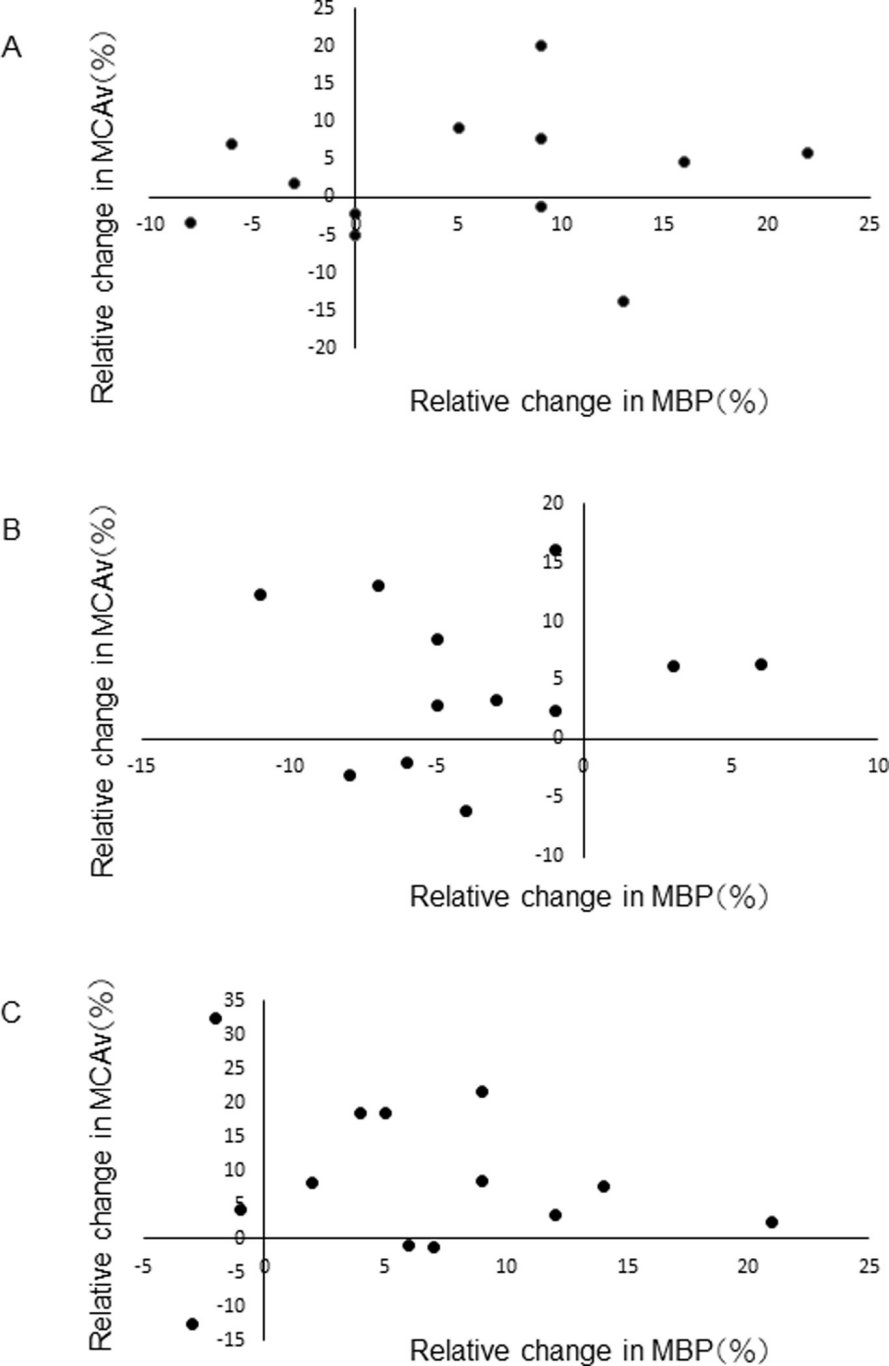
Relative change in individual MBP to relative change in individual MCAv. Individual relative changes in MBP are plotted against the relative change in MCAv. (A) an FS score, (B) an MS score and (C) a PR score. No significant correlation was observed.

Analysis of relative changes in MBP versus MCAv revealed no association between the two parameters for the FS, PS and MS scores (Fig 2).

## Discussion

The main finding of this study was a 5–9% increase in MCAv in subjects when playing an instrument, except when playing a score for the first time (i.e., FS). The increase was observed in most subjects. There were no significant concomitant increases in metabolic demand or ventilation. These results suggest that playing an instrument increases CBF with no observable increase in metabolic demand.

The extent of the increase in MCAv was less than that previously been reported to occur during physical exercise. The degree of increase in MCAv during exercise ranges from 10% to 20%, with MCAv increasing by 20% when *V*O_2_ increases to four times the resting level [5]. During exercise, MCAv continues to increase until *V*O_2_ reaches 70% of its maximum [14–16]. In the present study, the extent of increase in CBF associated with playing a musical instrument was much lower than that observed during exercise. CBF increased during instrumental playing, even though there was no increase in *V*O_2_. Therefore, the increase in MCAv during musical instrument playing was not due to metabolic processes.

There are two possible explanations for the lack of increase in MCAv in the FS trial. First, because of the difficulty inherent in playing music for the first time, the subjects chose music with a much lower level of difficulty than would be appropriate for their skills. For example, a subject who played Frederic Chopin’s *Heroic Polonaise* (Op. 53 in A-flat major) on the MS test played Gustav Lange’s Heather Rose (Op. 78, No. 3 in C major) on the FS test. According to the Japanese music publisher ZEN-ON MUSIC, *Heroic Polonaise* is ranked F (advanced upper) and *Heather Rose* is ranked A (beginner). Since they were professional musicians, the subjects in the present study would have found the FS score easy to perform. When a professional pianist requires bimanual coordination, the recruitment of motor networks in the brain is lower than that for non-musicians [1]. The subjects could perform complex bimanual movements more easily, which may be related to a reduced activity in the brain, possibly resulting in a subtle increase in MCAv. Second, the frequency of finger tapping was low because the subjects chose an easier FS music score. The frequency of fingering of both hands increases with increasing difficulty of the music. A relative increase in blood volume in the primary motor cortex observed by near-infrared spectroscopy was much larger in a maximum-effort finger-tapping task than in a low-frequency finger-tapping task [17]. The low frequency of finger tapping that occurs when playing an FS score may result in only a small degree of regional brain activation.

The MCAv measurements made during playing a musical instrument in the present study have two advantages over previous studies that have demonstrated activation in various brain regions. First, we targeted a large blood vessel and measured absolute changes in blood flow velocity. Studies using fMRI and PET have examined the occurrence of increases in blood volume in target regions of the brain relative to other brain regions while playing a musical instrument [1–4]. We have confirmed an increase in blood flow velocity in absolute value. Second, in the present study the subjects played their instruments under conditions close to those of a normal performance environment, whereas in previous fMRI and PET studies the subjects played fake instruments in a restricted environment. For example, a keyboard compatible with performing fMRI has been applied, but the subjects used only one hand to play it and/or only music scales were played rather than a full music score, subjects played their instrument while in a supine position, and the performance was done in loud extraneous noise conditions from fMRI and PET devices [18, 19]. These factors are likely to have affected the subjects’ performance and brain neural activity. The findings of the present study are more likely to reflect the normal CBF response to playing music, since the subjects were able to play their instrument in a normal playing environment.

The physiological factors underlying the increased MCAv observed during playing are controversial. Arterial pressure, PaCO_2_, autoregulation, neural activity and metabolic relate all impact MCAv [20]. The increase in MBP may contribute to the increase in MCAv observed in the present study since there was no increase in CI, which reflects vasodilatation. However, its contribution to MCAv was not a clear and at least non-linear, since no relationship was shown in Fig 2. P_ET_CO_2_ did not change and thus did not contribute to the increase in MCAv. The contribution of MBP and vasodilatation to the increase in MCAv was not simple nor clear.

MCAv involves blood flow to regions relating to instrumental performance. Instrumental performance involves sensory, motor, and auditory senses, which are distributed throughout the brain as integrated regions [4, 7]. This network consists of the dorsolateral, inferior frontal cortex (including Broca’s area), superior temporal gyrus (Wernicke’s area), marginal superior gyrus, supplementary motor area, and premotor area [18]. All of these sites receive blood via the MCA. Thus the MCAv in the present study reflects blood flow in these regions.

The present study did not provide consistent results for increases in MCAv and MBP. This is partly due to the fact that the music performed were not strictly controlled in nature. The subjects themselves selected the music according to the difficulty level, since the subjects’ performance abilities varied. Replacing it with exercise, the intensity of the exercise was not steady and uniform. The degree of increase in MCAv and MBP varies with exercise intensity [21]. When the increase in MCAv and MBP accompanied with playing musical instruments is examined in terms of hand and arm exercises, it is necessary to match the music for the trials. In turn, we successfully provided the responses in all tasks, implying roles of visual processing of reading music score, and the act of listening to the sound, the motor activity of playing. Some kind of technique is needed to clearly describe effects of playing a musical instrument on MCAv and MBP.

MCAv as measured by TCD has been evaluated previously as an index of CBF. MCAv can be treated as CBF when the diameter of the artery does not change. It has been reported that the MCA diameter measured during craniotomy or by nuclear magnetic resonance imaging does not change when physiological stimuli (e.g. arterial BP and PaCO_2_) are applied [22–24]. Even during exercise, changes in MCAv measured by TCD are comparable to changes in mean blood flow in the upstream artery, the internal carotid artery [5]. It is therefore reasonable to assume that MCAv indicates CBF in the present study.

Control data were not collected in this study so as to reduce the time burden on the subjects. However, given the early recovery of most variables back to the resting recorded before playing, it is reasonable to conclude that the observed changes were true responses to playing a musical instrument.

A limitation for this study is that Playing violin and piano are different in motor commitment. Further investigation using a larger sample size solely in violin is needed for a more definitive clarification.

## Conclusions

Playing a musical instrument increases MCAv, at least when complex music was played, with the degree of increase being partially dependent on the difficulty of the piece being played. Previous studies using fMRI and PET have revealed regions of increased blood flow in the brain when playing a musical instrument; however, these studies did not assess CBF in the whole brain. To the best of our knowledge, this is the first study to find a clear impact of playing a musical instrument on CBF.

## Abbreviations

BP: Blood pressure
CBF: cerebral blood flow
Cl: conductance index
fMRI: functional magnetic resonance imaging
FS: play for the first time
MBP: mean blood pressure
MCA: middle cerebral artery
MCAv: middle cerebral artery blood velocity
MS: already mastered
PaCO_2_: partial pressure of arterial carbon dioxide
PET: positron-emission topography
P_ET_CO_2_: end-tidal CO2 partial pressure
PR: music in practice
TCD: transcranial doppler
*V*O_2_: oxygen consumption
